# Supplementary material for: FERN – a Java framework for stochastic simulation and evaluation of reaction networks
Source: BMC Bioinformatics. 2008 Aug 29;9:356. doi: 10.1186/1471-2105-9-356 (PMC2553347; doi:10.1186/1471-2105-9-356)
Supplement: Additional file 1 — FERN distribution, Version 1.3. This archive contains the FERN source code and binaries as well as documentation and example models in FernML and SBML. [file 1471-2105-9-356-S1.zip › fern/doc/javadoc/index-files/index-7.html]

 
 
 
 
 
 
G-Index
 

 

 

 
function windowTitle()
{
    if (location.href.indexOf('is-external=true') == -1) {
        parent.document.title="G-Index";
    }
}
 
 
 

 

 
 


 
   
  
 
 
 
   
 
   
          Overview     
        Package   
        Class   
        Use   
          Tree     
          Deprecated     
       Index    
          Help     
   
 
 
  
 
 
 

 
  
   PREV LETTER   
   NEXT LETTER    
  
    FRAMES     
   NO FRAMES     
  
    All Classes  ');
  }
  //-->
 
 
    All Classes  
 


  
 
 
  
 

 A   B   C   D   E   F   G   H   I   J   L   M   N   O   P   R   S   T   U   V   
    
 G  
 
   get()   - 
Method in class fern.analysis. IntQueue 
  
   get()   - 
Method in interface fern.analysis. IntSearchStructure 
  
   get()   - 
Method in class fern.analysis. IntStack 
  
   getAdditionalAxesIterator()   - 
Method in class fern.tools.gnuplot. Axes 
 Iterator for the attached  Axes  objects.
   getAdditionalReactionNeighbors(int)   - 
Method in interface fern.analysis. NetworkSearchAction 
 Returns an iterator for additional neighbors of this species
   getAdditionalSpeciesNeighbors(int)   - 
Method in interface fern.analysis. NetworkSearchAction 
 Returns an iterator for additional neighbors of this reaction
   getAmount(int)   - 
Method in class fern.network. AmountManager 
 Gets the current amount of a species.
   getAmount(int)   - 
Method in class fern.simulation. Simulator 
 Gets the amount of the given species.
   getAmountBottomColor()   - 
Method in class fern.cytoscape. ColorCalculator 
  
   getAmountManager()   - 
Method in class fern.network. AbstractNetworkImpl 
  
   getAmountManager()   - 
Method in class fern.network.modification. CatalysedNetwork 
  
   getAmountManager()   - 
Method in class fern.network.modification. ExtractSubNetwork 
 Gets the   AmountManager   for the extracted subnet.
   getAmountManager()   - 
Method in class fern.network.modification. ModifierNetwork 
 Gets the   AmountManager   of the original network.
   getAmountManager()   - 
Method in class fern.network.modification. ReversibleNetwork 
 Gets the   AmountManager   for the modified network.
   getAmountManager()   - 
Method in interface fern.network. Network 
 Gets the   AmountManager   for this network.
   getAmountManager()   - 
Method in class fern.simulation. Simulator 
 Gets the   AmountManager  .
   getAmountTopColor()   - 
Method in class fern.cytoscape. ColorCalculator 
  
   getAnnotationManager()   - 
Method in class fern.network. AbstractNetworkImpl 
  
   getAnnotationManager()   - 
Method in class fern.network.modification. CatalysedNetwork 
  
   getAnnotationManager()   - 
Method in class fern.network.modification. ExtractSubNetwork 
 Gets an   AnnotationManager   for the redirected index space.
   getAnnotationManager()   - 
Method in class fern.network.modification. ModifierNetwork 
 Gets the   AnnotationManager   of the original network
   getAnnotationManager()   - 
Method in class fern.network.modification. ReversibleNetwork 
 Gets the   AnnotationManager   for the modified network.
   getAnnotationManager()   - 
Method in interface fern.network. Network 
 Gets the   AnnotationManager   for this network.
   getAsDouble(String)   - 
Method in class fern.tools. ConfigReader 
 Gets the value of the line  key=value  as double
   getAsInt(String)   - 
Method in class fern.tools. ConfigReader 
 Gets the value of the line  key=value  as int
   getAsString(String)   - 
Method in class fern.tools. ConfigReader 
 Gets the value of the line  key=value  as string
   getAsStringArr(String)   - 
Method in class fern.tools. ConfigReader 
 Gets the value of the line  key=value  as string array (the value is simply splitted by ,)
   getAttributeKeys()   - 
Method in class fern.cytoscape.ui. ExtendedPane 
  
   getAutocatalyticReactions()   - 
Method in class fern.analysis. AutocatalyticNetworkDetection 
 Gets the autocatalytic reactions as   BitVector  .
   getAutocatalyticSpecies()   - 
Method in class fern.analysis. AutocatalyticNetworkDetection 
 Gets the autocatalytic species as   BitVector  .
   getAvailableTypes()   - 
Static method in class fern.network. NetworkLoader 
 Gets types of networks that are readable by FERN.
   getAxes()   - 
Method in class fern.tools.gnuplot. GnuPlot 
 Returns the Axes object containing the data, labels and styles.
   getBinom(int, double)   - 
Method in class fern.tools. Stochastics 
 Gets a random number from the binomial distribution.
   getCatalysts(int)   - 
Method in class fern.network.creation. AutocatalyticNetwork 
 Implementation for the   CatalystIterator  .
   getCatalysts(int)   - 
Method in interface fern.network.creation. CatalystIterator 
 Gets the indices of the catalysts of a reaction.
   getCatalyzedKineticConstant()   - 
Method in class fern.network.creation. AutocatalyticNetwork 
 Gets the kinetic constant for catalyzed reactions.
   getColor(double, double)   - 
Method in class fern.cytoscape. ColorCalculator 
  
   getCommands()   - 
Method in class fern.tools.gnuplot. GnuPlot 
 Returns the list of commands, e.g. if you want to remove some command from it.
   getConstant(int)   - 
Method in class fern.network. AbstractKineticConstantPropensityCalculator 
 Gets the constant for a reaction
   getConstant(int)   - 
Method in class fern.network. ArrayKineticConstantPropensityCalculator 
  
   getConstantBySettingReactantsToStoich(Network, int)   - 
Static method in class fern.tools. NetworkTools 
 Gets the reaction rate constant by setting the reactant amounts to the stoichiometric
 coefficient.
   getConstantFromDeterministicRateConstant(double, int, double)   - 
Method in class fern.network. AbstractKineticConstantPropensityCalculator 
 Calculates the specific reaction probability rate constant c from the conventional
 deterministic rate constant k in some fixed volume v by the formula
 c=|reactants| !
   getContentAsArray(BitVector)   - 
Static method in class fern.tools. NumberTools 
 Gets the indices of the set bits of the given   BitVector   as array.
   getContentAsBitVector(int[])   - 
Static method in class fern.tools. NumberTools 
 Gets the content of the given array as   BitVector  , which means, that
 the  BitVector  will have a size equal to the maximal value in the 
 array and contains a 1 for each element which is contained at least once in the array
   getController(int)   - 
Method in class fern.benchmark. SimulatorCorrectness 
 Returns the   SimulationController   for the base class.
   getController(int)   - 
Method in class fern.benchmark. SimulatorPerformance 
 Extending classes have to determine the   SimulationController   of each
  Simulator  here.
   getController(int)   - 
Method in class fern.benchmark. SimulatorRandomNumbers 
 Returns the   SimulationController   for the base class.
   getController(int)   - 
Method in class fern.benchmark. SimulatorTime 
 Returns the   SimulationController   for the base class.
   getCounts()   - 
Method in class fern.tools. Stochastics 
 Gets the number of random number generations for each distribution (same order as in
   Stochastics.getNames()   since the last call of  resetCounts .
   getData()   - 
Method in class fern.tools.gnuplot. GnuPlot 
 Returns the data in gnuplot data file format as list containing the data for each added axes.
   getDefaultStyle()   - 
Method in class fern.tools.gnuplot. GnuPlot 
 The defaultStyle is used, when no style is defined for an column in an axes object.
   getDependent(int)   - 
Method in class fern.simulation.algorithm. DependencyGraph 
 Gets the descendants of a reaction in the dependency graph
   getEdgeClassifier()   - 
Method in class fern.cytoscape. NetworkChecker 
  
   getEntityValue(int)   - 
Method in class fern.simulation.observer. AmountIntervalObserver 
 Gets the actual amount of a species.
   getEntityValue(int)   - 
Method in class fern.simulation.observer. IntervalObserver 
 Gets the actual value of the entity.
   getEntityValue(int)   - 
Method in class fern.simulation.observer. MultiAmountIntervalObserver 
 Gets the actual amount of a species.
   getEntityValue(int)   - 
Method in class fern.simulation.observer. ReactionIntervalObserver 
 Gets the actual number of firings of a reaction since the simulation started.
   getEpsilon()   - 
Method in class fern.cellDesigner.ui. ExtendedPane 
  
   getEpsilon()   - 
Method in class fern.cytoscape.ui. ExtendedPane 
  
   getEpsilon()   - 
Method in class fern.example. HistogramDistanceTestSet 
  
   getEpsilon()   - 
Method in class fern.simulation.algorithm. AbstractBaseTauLeaping 
 The epsilon is an error control parameter and bounds the expected change of the 
 propensity functions by epsilon * a_sum.
   getExponential(double)   - 
Method in class fern.tools. Stochastics 
 Gets a random number from the exponential distribution.
   getFile()   - 
Method in class fern.example. HistogramDistanceTestSet 
  
   getFinalValue(int)   - 
Method in class fern.simulation.observer. IntervalObserver 
 Gets the last value of the given entity.
   getFoodSpecies()   - 
Method in class fern.analysis. AutocatalyticNetworkDetection 
 Gets the food molecules of the network.
   getGlobalParameters()   - 
Method in class fern.cellDesigner. CellDesignerPropensityCalculator 
 Gets the global parameters.
   getGlobalParameters()   - 
Method in class fern.network.sbml. SBMLPropensityCalculator 
 Gets the global parameters.
   getGrayScaleSpectrum(float)   - 
Static method in class fern.tools. ColorSpectrum 
 Gets a gray scale color representing the given number, 0 means white, 1 means black.
   getHistoAsParallelArray()   - 
Method in class fern.example. HistogramDistanceTestSet 
  
   getHistogram()   - 
Method in class fern.example. HistogramDistanceTestSet 
  
   getHistogram(int)   - 
Method in class fern.simulation.observer. AmountAtMomentObserver 
 Gets the histogram of the distribution for a species
   getHistogramAsString(Map<Integer, Integer>)   - 
Static method in class fern.tools. NumberTools 
 Gets a string representation of the histogram map.
   getImage()   - 
Method in class fern.tools.gnuplot. GnuPlot 
 Return the plotted data as image.
   getInitialAmount(int)   - 
Method in class fern.cellDesigner. CellDesignerNetworkWrapper 
  
   getInitialAmount(int)   - 
Method in class fern.cytoscape. CytoscapeNetworkWrapper 
  
   getInitialAmount(int)   - 
Method in class fern.network.creation. AutocatalyticNetwork 
  
   getInitialAmount(int)   - 
Method in class fern.network.fernml. FernMLNetwork 
  
   getInitialAmount(int)   - 
Method in class fern.network.modification. ExtractSubNetwork 
  
   getInitialAmount(int)   - 
Method in class fern.network.modification. ModifierNetwork 
  
   getInitialAmount(int)   - 
Method in interface fern.network. Network 
 Gets the initial amount of the specified molecule species.
   getInitialAmount(int)   - 
Method in class fern.network.sbml. SBMLNetwork 
  
   getInstance()   - 
Static method in class fern.tools. Stochastics 
 Singleton pattern method.
   getKey(int)   - 
Method in class fern.simulation.algorithm. IndexedPriorityQueue 
 Gets the tau of a reaction by lookup in the index
   getKineticLawSpecies(int)   - 
Method in class fern.cellDesigner. CellDesignerPropensityCalculator 
  
   getKineticLawSpecies(int)   - 
Method in interface fern.network. ComplexDependenciesPropensityCalculator 
 Gets the indices of the species that are included in the calculation of 
 the given reaction.
   getKineticLawSpecies(int)   - 
Method in class fern.network.sbml. SBMLPropensityCalculator 
  
   getLabel(String)   - 
Method in class fern.simulation.observer. Observer 
 Formats a label with the actual label format:
 %l is replaced by the passed argument
 %a is replaced by the simulator's name
 %n is replaced by the network's name
 
   getLabel(int)   - 
Method in class fern.tools.gnuplot. Axes 
 Gets the label for a column.
   getLabelFormat()   - 
Method in class fern.simulation.observer. Observer 
 Gets the label format of   Observer.getLabel(String)  .
   getLangevinThreshold()   - 
Method in class fern.simulation.algorithm. AbstractBaseTauLeaping 
 The Langevin threshold determines, when the normal distribution is used as an
 approximation for the poisson distribution.
   getMathTree(int)   - 
Method in class fern.network.sbml. SBMLPropensityCalculator 
 Gets the internal representation of the sbml kinetic law.
   getMin()   - 
Method in class fern.simulation.algorithm. IndexedPriorityQueue 
 Gets the index of the minimal tau in the queue.
   getMinKey()   - 
Method in class fern.simulation.algorithm. IndexedPriorityQueue 
 Gets the minimal tau in the queue.
   getMonomerAmount()   - 
Method in class fern.network.creation. AutocatalyticNetwork 
 Gets the initial amount of the monomers for a simulation algorithm.
   getName()   - 
Method in class fern.network. AbstractNetworkImpl 
  
   getName()   - 
Method in class fern.network.modification. ModifierNetwork 
 Gets the name of the original network
   getName()   - 
Method in interface fern.network. Network 
 Gets an identifier of the network.
   getName()   - 
Method in class fern.simulation.algorithm. AbstractBaseTauLeaping 
 Returns the name of this simulator
   getName()   - 
Method in class fern.simulation.algorithm. GibsonBruckSimulator 
  
   getName()   - 
Method in class fern.simulation.algorithm. GillespieEnhanced 
  
   getName()   - 
Method in class fern.simulation.algorithm. GillespieSimple 
  
   getName()   - 
Method in class fern.simulation.algorithm. HybridMaximalTimeStep 
  
   getName()   - 
Method in class fern.simulation.algorithm. TauLeapingAbsoluteBoundSimulator 
  
   getName()   - 
Method in class fern.simulation.algorithm. TauLeapingRelativeBoundSimulator 
  
   getName()   - 
Method in class fern.simulation.algorithm. TauLeapingSpeciesPopulationBoundSimulator 
  
   getName()   - 
Method in class fern.simulation. Simulator 
 Gets the name of the algorithm.
   getNames()   - 
Method in class fern.tools. Stochastics 
 Gets the names of the built-in distributions.
   getNc()   - 
Method in class fern.cellDesigner.ui. ExtendedPane 
  
   getNc()   - 
Method in class fern.cytoscape.ui. ExtendedPane 
  
   getNCritical()   - 
Method in class fern.simulation.algorithm. AbstractBaseTauLeaping 
 The nCritical determines, when a reaction is called critical.
   getNet()   - 
Method in class fern.simulation. Simulator 
 Gets the simulation network.
   getNetwork()   - 
Method in class fern.cellDesigner.ui. MainFrame 
  
   getNetwork()   - 
Method in class fern.cytoscape.ui. MainFrame 
  
   getNetworkAnnotation(String)   - 
Method in class fern.cytoscape. CytoscapeAnnotationManager 
  
   getNetworkAnnotation(String)   - 
Method in interface fern.network. AnnotationManager 
 Gets the network annotation of the specified field.
   getNetworkAnnotation(String)   - 
Method in class fern.network. AnnotationManagerImpl 
  
   getNetworkAnnotation(String)   - 
Method in class fern.network.fernml. FernMLAnnotationManager 
  
   getNetworkAnnotationTypes()   - 
Method in class fern.cytoscape. CytoscapeAnnotationManager 
  
   getNetworkAnnotationTypes()   - 
Method in interface fern.network. AnnotationManager 
 Gets the names of the network reaction.
   getNetworkAnnotationTypes()   - 
Method in class fern.network. AnnotationManagerImpl 
  
   getNetworkAnnotationTypes()   - 
Method in class fern.network.fernml. FernMLAnnotationManager 
  
   getNetworkChecker()   - 
Method in class fern.cytoscape. CytoscapeNetworkWrapper 
  
   getNetworkChecker()   - 
Method in class fern.cytoscape.ui. MainFrame 
  
   getNetworkViewObject()   - 
Method in class fern.cytoscape. CytoscapeNetworkWrapper 
  
   getNextThetaEvent()   - 
Method in class fern.simulation. Simulator 
 Theta defines a moment, where the simulator has to invoke  theta  of
 a observer.
   getNodeAttributeObject()   - 
Method in class fern.cytoscape. CytoscapeNetworkWrapper 
  
   getNodeClassifier()   - 
Method in class fern.cytoscape. NetworkChecker 
  
   getNodeParameter()   - 
Method in class fern.cytoscape. NetworkChecker 
  
   getNormal()   - 
Method in class fern.tools. Stochastics 
 Gets a random number from the Normal distribution
   getNormal(double, double)   - 
Method in class fern.tools. Stochastics 
 Gets a random number from the Normal distribution with given mean and stddev.
   getNum()   - 
Method in class fern.cellDesigner.ui. ExtendedPane 
  
   getNum()   - 
Method in class fern.cytoscape.ui. ExtendedPane 
  
   getNumAdditionalColumns()   - 
Method in class fern.tools.gnuplot. Axes 
 Gets the number of columns of the attached  Axes  objects.
   getNumbersTo(int)   - 
Static method in class fern.tools. NumberTools 
 Gets the numbers from 0 to n in an array.
   getNumBins()   - 
Method in class fern.benchmark. Benchmark 
 Gets the number of bins that are used for creating gnuplot histograms.
   getNumColumns()   - 
Method in class fern.tools.gnuplot. ArrayMatrixAxes 
  
   getNumColumns()   - 
Method in class fern.tools.gnuplot. Axes 
 Gets the number of columns.
   getNumColumns()   - 
Method in class fern.tools.gnuplot. CollectionAxes 
  
   getNumColumns()   - 
Method in class fern.tools.gnuplot. TransposedArrayMatrixAxes 
  
   getNumLeaps()   - 
Method in class fern.simulation.observer. LeapObserver 
 Gets the number of the performed leaps
   getNumMonomers()   - 
Method in class fern.network.creation. AutocatalyticNetwork 
 Gets if the number of monomers
   getNumReactions()   - 
Method in class fern.network. AbstractNetworkImpl 
  
   getNumReactions()   - 
Method in class fern.network.fernml. FernMLNetwork 
  
   getNumReactions()   - 
Method in class fern.network.modification. CatalysedNetwork 
  
   getNumReactions()   - 
Method in class fern.network.modification. ExtractSubNetwork 
 Gets the number of reactions in the extracted subnet.
   getNumReactions()   - 
Method in class fern.network.modification. ModifierNetwork 
 Gets the number of reaction in the original network.
   getNumReactions()   - 
Method in class fern.network.modification. ReversibleNetwork 
 Gets the number of reactions in the modified network (which is 2*number of reaction
 in the original network).
   getNumReactions()   - 
Method in interface fern.network. Network 
 Gets the number of reaction within the network.
   getNumRows()   - 
Method in class fern.tools.gnuplot. ArrayMatrixAxes 
  
   getNumRows()   - 
Method in class fern.tools.gnuplot. Axes 
 Gets the number of rows.
   getNumRows()   - 
Method in class fern.tools.gnuplot. CollectionAxes 
  
   getNumRows()   - 
Method in class fern.tools.gnuplot. TransposedArrayMatrixAxes 
  
   getNumRuns()   - 
Method in class fern.cellDesigner.ui. OverviewPane 
  
   getNumRuns()   - 
Method in class fern.cytoscape.ui. OverviewPane 
  
   getNumSimpleCalls()   - 
Method in class fern.simulation.algorithm. AbstractBaseTauLeaping 
 The numSimpleCalls determines, how often the SSA is called, when tau leaping is
 abandoned.
   getNumSimulations()   - 
Method in class fern.simulation.observer. Observer 
 Gets the number of finished repeats of the simulation.
   getNumSpecies()   - 
Method in class fern.network. AbstractNetworkImpl 
  
   getNumSpecies()   - 
Method in class fern.network.fernml. FernMLNetwork 
  
   getNumSpecies()   - 
Method in class fern.network.modification. ExtractSubNetwork 
 Gets the number of species in the extracted subnet.
   getNumSpecies()   - 
Method in class fern.network.modification. ModifierNetwork 
 Gets the number of species in  the original network.
   getNumSpecies()   - 
Method in interface fern.network. Network 
 Gets the number of species within the network.
   getNumSteps()   - 
Method in class fern.example. CellGrowthObserver 
 Gets the number of steps taken in one simulation
   getOriginalNetwork()   - 
Method in class fern.network.modification. ModifierNetwork 
 Gets the original network.
   getOriginalReaction(int)   - 
Method in class fern.network.modification. ExtractSubNetwork 
 Redirects a reaction index from the subnet index space to the original index space
   getOriginalSpecies(int)   - 
Method in class fern.network.modification. ExtractSubNetwork 
 Redirects a species index from the subnet index space to the original index space
   getOtherAmount()   - 
Method in class fern.network.creation. AutocatalyticNetwork 
 Gets the initial amount of the not-monomers for a simulation algorithm.
   getParentNetwork()   - 
Method in class fern.network.modification. ModifierNetwork 
 Gets the parent network.
   getPlotCommand(List<File>)   - 
Method in class fern.tools.gnuplot. GnuPlot 
 Returns the plot command for a given filename
   getPoisson(double)   - 
Method in class fern.tools. Stochastics 
 Gets a random number from the Poisson distribution.
   getPrintWriter()   - 
Method in class fern.simulation.observer. Observer 
 Gets the registered   PrintWriter  
   getProb(int, int)   - 
Method in class fern.tools.functions. Probability.Constant 
  
   getProb(int, int)   - 
Method in interface fern.tools.functions. Probability 
 Gets the probability depending on two integers.
   getProb(int, int)   - 
Method in class fern.tools.functions. Probability.ReactionProbability 
  
   getProducts(int)   - 
Method in class fern.network. AbstractNetworkImpl 
  
   getProducts(int)   - 
Method in class fern.network.modification. CatalysedNetwork 
  
   getProducts(int)   - 
Method in class fern.network.modification. ExtractSubNetwork 
 Gets the products of a reaction.
   getProducts(int)   - 
Method in class fern.network.modification. ModifierNetwork 
 Gets the products of a reaction in the original network.
   getProducts(int)   - 
Method in class fern.network.modification. ReversibleNetwork 
 Gets the products of a reaction
   getProducts(int)   - 
Method in interface fern.network. Network 
 Gets the products of the specified reaction.
   getPropensity(int)   - 
Method in class fern.simulation. Simulator 
 Gets the current propensity for the given reaction.
   getPropensityCalculator()   - 
Method in class fern.network. AbstractNetworkImpl 
  
   getPropensityCalculator()   - 
Method in class fern.network.modification. CatalysedNetwork 
  
   getPropensityCalculator()   - 
Method in class fern.network.modification. ExtractSubNetwork 
 Gets the   PropensityCalculator   for the extracted subnet.
   getPropensityCalculator()   - 
Method in class fern.network.modification. ModifierNetwork 
 Gets the   PropensityCalculator   of the original network.
   getPropensityCalculator()   - 
Method in class fern.network.modification. ReversibleNetwork 
 Gets the   PropensityCalculator   for the modified network.
   getPropensityCalculator()   - 
Method in interface fern.network. Network 
 Gets the   PropensityCalculator   for this network.
   getPropensityCalculator()   - 
Method in class fern.simulation. Simulator 
 Gets the   PropensityCalculator  .
   getRawData()   - 
Method in class fern.analysis. ShortestPath.Path 
 Returns the raw path: Is ends with a species index and is composed of alternating
 indices of species and reactions.
   getReactants(int)   - 
Method in class fern.network. AbstractNetworkImpl 
  
   getReactants(int)   - 
Method in class fern.network.modification. CatalysedNetwork 
  
   getReactants(int)   - 
Method in class fern.network.modification. ExtractSubNetwork 
 Gets the reactants of a reaction.
   getReactants(int)   - 
Method in class fern.network.modification. ModifierNetwork 
 Gets the reactants of a reaction in the original network.
   getReactants(int)   - 
Method in class fern.network.modification. ReversibleNetwork 
 Gets the reactants of a reaction
   getReactants(int)   - 
Method in interface fern.network. Network 
 Gets the reactants of the specified reaction.
   getReactionAnnotation(int, String)   - 
Method in class fern.cytoscape. CytoscapeAnnotationManager 
  
   getReactionAnnotation(int, String)   - 
Method in interface fern.network. AnnotationManager 
 Gets the reaction annotation of the specified field.
   getReactionAnnotation(int, String)   - 
Method in class fern.network. AnnotationManagerImpl 
  
   getReactionAnnotation(int, String)   - 
Method in class fern.network.fernml. FernMLAnnotationManager 
  
   getReactionAnnotationTypes(int)   - 
Method in class fern.cytoscape. CytoscapeAnnotationManager 
  
   getReactionAnnotationTypes(int)   - 
Method in interface fern.network. AnnotationManager 
 Gets the names of the species reaction.
   getReactionAnnotationTypes(int)   - 
Method in class fern.network. AnnotationManagerImpl 
  
   getReactionAnnotationTypes(int)   - 
Method in class fern.network.fernml. FernMLAnnotationManager 
  
   getReactionCoefficient(Node)   - 
Method in class fern.cytoscape. NetworkChecker.NodeParameter 
  
   getReactionColor()   - 
Method in class fern.cytoscape. ColorCalculator 
  
   getReactionName(int)   - 
Method in class fern.network. AbstractNetworkImpl 
  
   getReactionName(int)   - 
Method in class fern.network.modification. CatalysedNetwork 
  
   getReactionName(int)   - 
Method in class fern.network.modification. ExtractSubNetwork 
 Gets a string representation of the reaction
   getReactionName(int)   - 
Method in class fern.network.modification. ModifierNetwork 
 Gets a string representation of the reactio in the original network.
   getReactionName(int)   - 
Method in class fern.network.modification. ReversibleNetwork 
 Gets a string representation of the reaction in the modified network.
   getReactionName(int)   - 
Method in interface fern.network. Network 
 Gets a string representation of the reaction.
   getReactionNames(Network, int[])   - 
Static method in class fern.tools. NetworkTools 
 Transforms the reaction indices in the given array to the corresponding names.
   getReactionNameWithAmounts(Network, int...)   - 
Static method in class fern.tools. NetworkTools 
 Gets a string representation of the given reactions and the amounts of each participating species in parentheses.
   getReactions()   - 
Method in class fern.analysis. ShortestPath.Path 
 Returns all the reactions on this path.
   getReactionsOf(Network, String[])   - 
Static method in class fern.tools. NetworkTools 
 Gets the reaction whose products / reactants are in speciesName
   getReactionView(int)   - 
Method in class fern.cytoscape. CytoscapeNetworkWrapper 
  
   getRecentData()   - 
Method in class fern.simulation.observer. IntervalObserver 
 Gets the recorded data of the most recent simulation run.
   getRecordStart()   - 
Method in class fern.simulation.observer. ReactionIntervalObserver 
 Gets the moment in time where the recording starts
   getReversePropensityCalculator()   - 
Method in class fern.network.creation. AutocatalyticNetwork 
 Gets the  PropensityCalculator  which has to be used for instantiation
 of the   ReversibleNetwork  .
   getRoot()   - 
Method in class fern.network.sbml. MathTree 
 Gets the root of this MathTree.
   getSBMLModel()   - 
Method in class fern.network.sbml. SBMLNetwork 
 Gets the libsbml model.
   getScale()   - 
Method in class fern.cytoscape. ColorCalculator 
  
   getScaleMax()   - 
Method in class fern.cytoscape. ColorCalculator 
  
   getSeed()   - 
Method in class fern.tools. Stochastics 
 Gets the actual seed of the random number generator.
   getShowSteps()   - 
Method in class fern.benchmark. SimulatorPerformance 
 Gets the number of iterations between two  present -calls.
   getSimulator()   - 
Method in class fern.cellDesigner.ui. OverviewPane 
  
   getSimulator()   - 
Method in class fern.cytoscape.ui. OverviewPane 
  
   getSimulator()   - 
Method in class fern.example. HistogramDistanceTestSet 
  
   getSimulator()   - 
Method in class fern.simulation.observer. Observer 
 Gets the simulator
   getSimulators()   - 
Method in class fern.benchmark. SimulatorPerformance 
 Gets the simulators used by this benchmark.
   getSpecies()   - 
Method in class fern.analysis. ShortestPath.Path 
 Returns all the species on this path.
   getSpecies()   - 
Method in class fern.network.sbml. MathTree 
 Gets the species present in this tree.
   getSpeciesAnnotation(int, String)   - 
Method in class fern.cytoscape. CytoscapeAnnotationManager 
  
   getSpeciesAnnotation(int, String)   - 
Method in interface fern.network. AnnotationManager 
 Gets the species annotation of the specified field.
   getSpeciesAnnotation(int, String)   - 
Method in class fern.network. AnnotationManagerImpl 
  
   getSpeciesAnnotation(int, String)   - 
Method in class fern.network.fernml. FernMLAnnotationManager 
  
   getSpeciesAnnotationTypes(int)   - 
Method in class fern.cytoscape. CytoscapeAnnotationManager 
  
   getSpeciesAnnotationTypes(int)   - 
Method in interface fern.network. AnnotationManager 
 Gets the names of the species annotations.
   getSpeciesAnnotationTypes(int)   - 
Method in class fern.network. AnnotationManagerImpl 
  
   getSpeciesAnnotationTypes(int)   - 
Method in class fern.network.fernml. FernMLAnnotationManager 
  
   getSpeciesByName(String)   - 
Method in class fern.network. AbstractNetworkImpl 
  
   getSpeciesByName(String)   - 
Method in class fern.network.modification. ExtractSubNetwork 
 Gets the index of the species by its name.
   getSpeciesByName(String)   - 
Method in class fern.network.modification. ModifierNetwork 
 Gets the index of the species by its name in the original network.
   getSpeciesByName(String)   - 
Method in interface fern.network. Network 
 Gets the species index by name.
   getSpeciesIndices(Network, String[])   - 
Static method in class fern.tools. NetworkTools 
 Transforms the species names in the given array to the corresponding indices.
   getSpeciesInitialAmount(Node)   - 
Method in class fern.cytoscape. NetworkChecker.NodeParameter 
  
   getSpeciesMapping()   - 
Method in class fern.network. AbstractNetworkImpl 
 Gets the mapping from species names to their indices.
   getSpeciesName(int)   - 
Method in class fern.network. AbstractNetworkImpl 
  
   getSpeciesName(int)   - 
Method in class fern.network.modification. ExtractSubNetwork 
 Gets the name of the species with given index.
   getSpeciesName(int)   - 
Method in class fern.network.modification. ModifierNetwork 
 Gets the name of the species by index in the original network.
   getSpeciesName(int)   - 
Method in interface fern.network. Network 
 Gets the species name by index.
   getSpeciesNames(Network, int[])   - 
Static method in class fern.tools. NetworkTools 
 Transforms the species indices in the given array to the corresponding names.
   getSpeciesNameWithAmount(Network, int...)   - 
Static method in class fern.tools. NetworkTools 
 Gets the species names together with its actual amount in parentheses
   getSpeciesView(int)   - 
Method in class fern.cytoscape. CytoscapeNetworkWrapper 
  
   getSpectrum(float, Color...)   - 
Static method in class fern.tools. ColorSpectrum 
 Gets a color representing the given number in an given color scale.
   getStyle(int)   - 
Method in class fern.tools.gnuplot. Axes 
 Gets the style for a column.
   getStyles()   - 
Method in class fern.simulation.observer. AmountAtMomentObserver 
  
   getStyles()   - 
Method in class fern.simulation.observer. AmountIntervalObserver 
  
   getStyles()   - 
Method in class fern.simulation.observer. FireTypeObserver 
  
   getStyles()   - 
Method in interface fern.simulation.observer. GnuPlotObserver 
 Gets the styles for the columns.
   getStyles()   - 
Method in class fern.simulation.observer. LeapObserver 
  
   getStyles()   - 
Method in class fern.simulation.observer. MultiAmountIntervalObserver 
  
   getStyles()   - 
Method in class fern.simulation.observer. RandomNumberGeneratorCallObserver 
  
   getStyles()   - 
Method in class fern.simulation.observer. ReactionIntervalObserver 
  
   getTheta()   - 
Method in class fern.simulation.observer. Observer 
 Gets the moment in time, where the simulator has to invoke   Observer.theta(double)  .
   getThreshold()   - 
Method in class fern.cellDesigner.ui. ExtendedPane 
  
   getThreshold()   - 
Method in class fern.cytoscape.ui. ExtendedPane 
  
   getTime()   - 
Method in class fern.cellDesigner.ui. OverviewPane 
  
   getTime()   - 
Method in class fern.cytoscape.ui. OverviewPane 
  
   getTime()   - 
Method in class fern.simulation.controller. DefaultController 
 Gets the time where the simulation has to stop.
   getTime()   - 
Method in class fern.simulation. Simulator 
 Gets the actual simulation time.
   getTitlesCommand(String[])   - 
Method in class fern.simulation.observer. Observer 
 Gets a gnuplot command if you want to plot a histogram and have
 the data labels for each value in an array
   getTop(int)   - 
Method in class fern.simulation.algorithm. AbstractTauLeapingPropensityBoundSimulator 
  
   getTop(int)   - 
Method in class fern.simulation.algorithm. TauLeapingAbsoluteBoundSimulator 
  
   getTop(int)   - 
Method in class fern.simulation.algorithm. TauLeapingRelativeBoundSimulator 
  
   getTrendSpecies()   - 
Method in class fern.cellDesigner.ui. ExtendedPane 
  
   getTrendSpecies()   - 
Method in class fern.cytoscape.ui. ExtendedPane 
  
   getUncatalyzedKineticConstant()   - 
Method in class fern.network.creation. AutocatalyticNetwork 
 Gets the kinetic constant for not catalyzed reactions.
   getUnif()   - 
Method in class fern.tools. Stochastics 
 Gets a random number from the uniform distribution between 0 and 1.
   getUnif(int, int)   - 
Method in class fern.tools. Stochastics 
 Gets a uniformly chosen random number between  min  (inclusive) and  max  (exclusive)
   getUseSimpleFactor()   - 
Method in class fern.simulation.algorithm. AbstractBaseTauLeaping 
 The useSimpleFactor determines, when the tau leaping is abandoned and the SSA
 method is used (if tau   getV(int, int)   - 
Method in class fern.simulation.algorithm. AbstractBaseTauLeaping 
  
   getVolume()   - 
Method in class fern.simulation. Simulator 
 Gets the volume of the reaction network.
   GibsonBruckSimulator   - Class in  fern.simulation.algorithm  Implementation of the algorithm of Gibson and Bruck.   GibsonBruckSimulator(Network)   - 
Constructor for class fern.simulation.algorithm. GibsonBruckSimulator 
  
   GillespieEnhanced   - Class in  fern.simulation.algorithm  This is an enhanced version of the original Direct method developed by
 Gillespie.   GillespieEnhanced(Network)   - 
Constructor for class fern.simulation.algorithm. GillespieEnhanced 
  
   GillespieSimple   - Class in  fern.simulation.algorithm  Implementation of Gillespie's Direct method.   GillespieSimple(Network)   - 
Constructor for class fern.simulation.algorithm. GillespieSimple 
  
   GnuPlot   - Class in  fern.tools.gnuplot  The class  GnuPlot  provides methods for handling gnuplot data.   GnuPlot()   - 
Constructor for class fern.tools.gnuplot. GnuPlot 
 The constructor sets the DefaultCloseOperation of this JFrame to DISPOSE_ON_CLOSE.
   GnuPlotObserver   - Interface in  fern.simulation.observer  Interface for certain observer which can produce gnuplot data.   goOn(Simulator)   - 
Method in class fern.simulation.controller. AndController 
  
   goOn(Simulator)   - 
Method in class fern.simulation.controller. DefaultController 
  
   goOn(Simulator)   - 
Method in class fern.simulation.controller. OrController 
  
   goOn(Simulator)   - 
Method in interface fern.simulation.controller. SimulationController 
 Returns whether or not to go on with the given simulation
   goOn(Simulator)   - 
Method in class fern.simulation.observer. AmountAtMomentObserver 
 Causes the simulator to stop when  theta  has been passed and the amounts
 have been recorded.
 
 


 
   
  
 
 
 
   
 
   
          Overview     
        Package   
        Class   
        Use   
          Tree     
          Deprecated     
       Index    
          Help     
   
 
 
  
 
 
 

 
  
   PREV LETTER   
   NEXT LETTER    
  
    FRAMES     
   NO FRAMES     
  
    All Classes  ');
  }
  //-->
 
 
    All Classes  
 


  
 
 
  
 

 A   B   C   D   E   F   G   H   I   J   L   M   N   O   P   R   S   T   U   V
